# Supplementary material for: Lipid Differences and Related Metabolism Present on the Hand Skin Surface of Different-Aged Asiatic Females—An Untargeted Metabolomics Study
Source: Metabolites. 2023 Apr 13;13(4):553. doi: 10.3390/metabo13040553 (PMC10142664; doi:10.3390/metabo13040553)
Supplement: Supplementary file 1 [file metabolites-13-00553-s001.zip › metabolites-2240290-supplementary.pdf]

Supplementary Table S1. The detailed ion source conditions of quadrupole time-of-flight mass spectrometry.

Parameters	ESI+
Sample temperature	10 ℃
Column temperature	50 ℃
Analysis mode	sensitivity
Capillary voltage	3000 V
Ion source temperature	120 ℃
Desolvation temperature	500 ℃
Cone gas flow	50 L/h
Desolvation gas flow	900 L/h
